# SLAMF6 clustering is required to augment T cell activation

**DOI:** 10.1371/journal.pone.0218109

**Published:** 2019-06-14

**Authors:** Matthew A. Dragovich, Kieran Adam, Marianne Strazza, Anna S. Tocheva, Michael Peled, Adam Mor

**Affiliations:** 1 Columbia Center for Translational Immunology, Columbia University Medical Center, New York, New York, United States of America; 2 Division of Rheumatology, Columbia University Medical Center, New York, New York, United States of America; 3 Division of Pulmonary Medicine, Sheba Medical Center, Ramat Gan, Israel; Hungarian Academy of Sciences, HUNGARY

## Abstract

The signaling lymphocytic activation molecule (SLAM) family is comprised of nine distinct receptors that are expressed exclusively on hematopoietic cells. Most of these transmembrane receptors are homotypic by nature and downstream signaling occurs when cells that express the same SLAM receptor interact. Previous studies have determined that anti-SLAMF6 antibodies can have a therapeutic effect in autoimmunity and cancer. However, little is known about the role of SLAMF6 in the adaptive immune responses and in order to utilize SLAMF6 interventional approaches, a better understanding of the biology of this receptor in T cell is warranted. Accordingly, the objective of our study was to investigate both functionally and structurally the role of SLAMF6 in T cell receptor (TCR) mediated responses. Biochemical and genetic experiments revealed that SLAMF6 was required for productive TCR downstream signaling. Interestingly, SLAMF6 ectodomain was required for its function, but not for its recruitment to the immunological synapse. Flow-cytometry analysis demonstrated that tyrosine 308 of the tail of SLAMF6 was crucial for its ability to enhance T cell function. Imaging studies revealed that SLAMF6 clustering, specifically with the TCR, resulted in dramatic increase in downstream signaling. Mechanistically, we showed that SLAMF6 enhanced T cell function by increasing T cell adhesiveness through activation of the small GTPase Rap1. Taken together SLAMF6 is an important regulator of T cell activation where both its ectodomain and its endodomain contribute differentially to T cell functions. Additional studies are underway to better evaluate the role of anti-SLAMF6 approaches in specific human diseases.

## Introduction

The T cell receptor (TCR) is a complex of proteins found on the surface of T cells and is utilized to recognize antigens that are presented in the context of major histocompatibility complex (MHC) class I or class II molecules located on the surface of antigen presenting cells (APC) [1]. Along with the TCR-MHC complex, further signals in the form of co-receptors are required for proper T cell activation. For cytotoxic T cells the additional MHC class I ligating co-receptor is CD8. In the case of helper and regulatory T cells the co-receptor is CD4 which ligates MHC class II. In addition to TCR specific co-receptors, there are other co-receptors that are critical for T cell activation which do not associate directly with the MHC. Such co-receptors include CD28, CD40L and CD2. Upon ligation of these receptors, helper T cells can have a powerful increase in interleukin (IL)-2 secretion [2] and regulatory T cells experience an increase in IL-10 release [3]. However, *in-trans* physical engagement of these co-receptors with their ligands is not sufficient for proper immune responses. In the instance of CD28 and CD40L, clustering in the immunological synapse (IS), the interface between T cells and APC, is also a critical aspect of their function [4–6].

Signaling lymphocyte activation molecule 6 (SLAMF6) (Ly108 in mice, NTB-A or SF2000 in humans) is a homophilic receptor belonging to the superfamily immunoglobulin (Ig) domain-containing molecules. It is known to be widely and exclusively expressed on hematopoietic cells [7]. The structure of SLAMF6 is comprised of an amino terminal Ig like variable (V) domain and a membrane proximal constant 2 (C2) domain in the extracellular portion. The SLAMF6 intracellular portion is characterized by two immune receptor tyrosine-based switch motifs (ITSM) [8, 9] (S1 Fig). These ITSM act as binding sites for adaptor molecules such as SLAM adaptor protein (SAP) and Ewing sarcoma associated transcript (EAT-2) [7, 10, 11]. It has been suggested that SLAMF6-SLAMF6 complexes form in a *“head to head”* fashion via interacting IgV like domains [12]. SLAMF6 knock out mice experience a lack of germinal center formation and consequently, lack B cell education by T cells [13].

By the means of a phospho-proteomic analysis we have determined that during ligation of the TCR and programmed cell death 1, SLAMF6 ITSMs were differentially phosphorylated. Due to the fact that TCR engagement delivers the first signal for T cell activation, we postulated that SLAMF6 may play an important role in T cell activation in the context of the IS. Through a set of biochemical and functional experiments, we show here that SLAMF6 clustering is required for its stimulatory functions and that trafficking to the synapse is integral to that. A structure-function analysis revealed the contribution of both the SLAMF6 ectodomain and one of the ITSMs to its function as a potent co-stimulatory receptor promoting T activation, proliferation and adhesion.

## Materials and methods

### General reagents

RPMI 1640 medium, DMEM, Dulbecco’s PBS, and FBS were purchased from Life Technologies. Staphylococcus Enterotoxin E (SEE) was acquired from Toxin Technology. Puromycin was obtained from Sigma-Aldrich.

### Cell culture and transfection

All the cells were maintained in 5% CO_2_ at 37°C. Jurkat T cells and Raji B cells were obtained from the American Type Culture Collection and maintained in RPMI 1640 medium supplemented with 10% FBS and 100 U/ml penicillin and streptomycin. Primary CD3^+^ T cells were grown in RPMI 1640 medium supplemented with 10% FBS and 100 U/ml penicillin and streptomycin, non-essential amino acids and L-glutamine (2mM). HEK293T and U20s cells were obtained from the American Type Culture Collection cultured in DMEM media supplemented with 10% FBS and 100 U/ml penicillin and streptomycin. DNA expression constructs were introduced into the cells by nucleofection (Lonza) with efficiency of 30–60% and viability of 80–90%.

### Antibodies production and stimulation

Anti-CD3 (UCHT1) and PE conjugated anti-SLAMF6 (292811) were purchased from R&D Systems. Goat-anti-mouse IgG (Poly4053), Alexa Fluor 647 conjugated anti-ERK (4B11B69), phospho Thr202/Tyr204, Alexa Fluor 647 conjugated IgG2b iso-type control (MOPC-173), and anti-SLAMF6 (NT-7) were purchased from BioLegend. SLAMF6 (HA12OC3104) was purchased from Sino Biologics Inc. PE conjugated IgG2a iso-type control (eBR2a) was purchased from eBioscience. Western blot antibodies for pERK (E10) Thr202/Tyr204, pZAP70 (Y352) Tyr309, pAKT (L32A4) Thr308 and pSRC (Y527) were purchased from Cell Signaling. Anti-beta actin (AC-15) was purchased from Invitrogen. Western blot secondary antibodies IRDye 680RD Goat anti-mouse IgG, IRDye 800CW Goat anti-mouse IgG and IRDye 680RD Goat anti-rabbit IgG were purchased from Licor. Anti-SLAMF6 (NT-7) Fab fragments were generated using the Pierce Fab Preparation Kit obtained from Thermo Fisher. Cells were stimulated with the following soluble antibodies: anti-CD3/anti-mouse IgG (3.25 μg/mL), anti-SLAMF6 (3.25 μg/mL), anti-mouse IgG (1.63 μg/mL), anti-SLAMF6 *Fab* (3.25 μg/mL) anti-mouse IgG (1.63 μg/mL). Cells were stimulated with the following immobilized antibodies: anti-CD3/anti-mouse IgG (1.5 μg/mL), anti-SLAMF6 (5 μg/mL).

### DNA constructs

SLAMF6-GFP fusion expression constructs were generated through PCR amplification and cloning of SLAMF6 (DNASU) into pEGFP-N1 vector (Invitrogen). SLAMF6 Y284F GFP and SLAMF6 Y308F GFP were generated via site directed mutation performed with the Agilent Technologies QuickChange Lightning Site-Directed Mutagenesis kit. ΔSLAMF6 GFP was generated by cloning of the residues 1–15 of the gene PAG and placing it before the residues 207–332 of SLAMF6 in the SLAMF6-GFP fusion expression construct.

### Knocking down and knocking out SLAMF6

SLAMF6 was stably knocked down in Jurkat T cells by RNA interference using Mission shRNA plasmids (Sigma-Aldrich). Lentiviral particles were generated by transfecting HEK293T cells with pMD2G, psPAX2, and the shRNA plasmid using SuperFect (Qiagen). T cells were transduced by centrifugation and selected with puromycin. SLAMF6 was knocked out in Jurkat T cells by CRISPR-Cas9 using two of the lentiCRISPR v2 plasmid purchased from GeneScript. Both different plasmids each containing a unique guide RNA were used. Two sets of lentiviral particles were then generated as before each set containing one of the different lentiCRISPR v2 plasmids. Both sets of viral particles were transduced by centrifugation in to the same group of cells which were selected with puromycin. Deletion of the gene was validated by flow cytometry.

### Western blot analysis

Cell lysates were created via radio immunoprecipitation assay (RIPA) buffer and were separated by Tris-glycine PAGE, semi-dry transferred to nitrocellulose membranes and visualized using the Licor CLX imaging system. Unaltered complete blots are provided as a supplement with cropping regions indicated. Bands outside of the cropping region are considered non-specific due to their random appearances when conditions were repeated independently.

### Rap1 activation assay

Activated Rap1 was detected with a GST pull-down assay, as previously described [14]. Jurkat T cells were stimulated with αCD3 for 2 min before being lysed. Cell lysates were incubated at 4°C for 1 hour with GST-RBD-RalGDS coupled to glutathione beads to pull down activated Rap1. The pull-down lysates were then separated and transferred as described above. Blots were then stained with αRap1 and immunoreactive bands were visualized using the Licor CLX imaging system.

### Flow-cytometry

To verify the SLAMF6 expression, Jurkat T cells were stained with fluorescently conjugated antibodies specific for SLAMF6 in FACS Buffer, then washed before the events were recorded using a FACSCalibur (BD Biosciences). Cells were stimulated with anti-CD3, fixed in 4% paraformaldehyde and permeabilized with 100% methanol. Next, cells were stained with fluorescently conjugated Abs specific for anti-pERK, phospho Thr202/Tyr204 and events were recorded using a FACS Canto (BD Biosciences). All data was analyzed using FlowJo software.

### Conjugate formation assay

SEE (Toxin Technology) loaded Raji B cells were mixed with Jurkat T cells and plated on 35-mm glass bottom culture plates and incubated for 45 min prior to imaging. Images were taken with Zeiss LSM 800 confocal microscope and quantified for conjugate formation.

### Primary T cell Isolation

RosetteSep negative selection human T cell enrichment cocktail (Stem Cell Technologies) was added to whole blood extracted from three individual patients at a ratio of 5:100 for 20 min. The blood was then subjected to ficoll gradient separation via lymphoprep (Stem Cell Technologies). The CD3^+^ cells were harvested and used for experiments the same day.

### The enzyme-linked immunosorbent assay (ELISA)

To determine the concentration of secreted proteins after stimulation a human IFN-γ or IL-2 kit from BioLegend was used according to the manufacturer’s protocols. Cells were stimulated as indicated.

### Cell proliferation assay

Jurkat T cells were activated with soluble αCD3 or αCD3/αSLAMF6 and cultured for 72 hrs. Number of cells was assessed by automated counting (Invitrogen Countess II) in the presence of trypan blue. Primary CD3^+^ T cells were isolated, stained with 1μM of Carboxyfluorescein succinimidyl ester (CFSE) then activated with immobilized αCD3 or αCD3/αSLAMF6 for 120 hrs. Cells were then assayed for proliferation via flow cytometry after a period of five days under stimulatory conditions.

### Proximity ligation assay

The proximity ligation (PLA) kit was obtained from Sigma-Aldrich. Millicell 8-well glass slide plates were coated with poly-L-lysine for 1 hr. at 5% CO_2_ and 37°C. Jurkat T cells were then added to the slide and allowed to adhere for 1 hr. PMA (0.1μg/mL) Ionomycin (1μg/mL) was used to stimulate the cells for 5 min at 5% CO_2_ and 37°C. Primary T cells were stimulated with immobilized antibodies for 120 hrs. Cells were then subjected to the manufactures protocol for PLA. Images were acquired using a Zeiss Imager.

### Adhesion assay

U2Os osteosarcoma epithelial cells were stably transfected with SLAMF6 and VCAM-1 by lentiviral transduction as described above, selected in blasticidin and further sorted via flow-cytometry. SLAMF6 KO Jurkat T cells were transiently transfected with GFP control, SLAMF6 GFP, SLAMF6 Y285F GFP, SLAMF6 Y309F GFP or ΔSLAMF6 GFP and co-cultured with the U20s cells expressing SLAMF6 and VCAM-1 at 5% CO_2_ and 37°C for 20 min in the presence of αCD3 in an adhesion buffer consisting of PBS 0.5% BSA 2mM MgCl_2_ 1mM CaCl_2_. The cells were then washed off by vigorously pipetting. The amount of GFP positive cells was quantified before and after washing on a Nikon Ti Eclipse.

### Statistics

Values are reported as means SEM. All statistical analyses were performed using student *t*-test in using GraphPad Prism (version 8.0).

## Results

### SLAMF6 is a stimulatory T cell co-receptor

**Fig 1:**
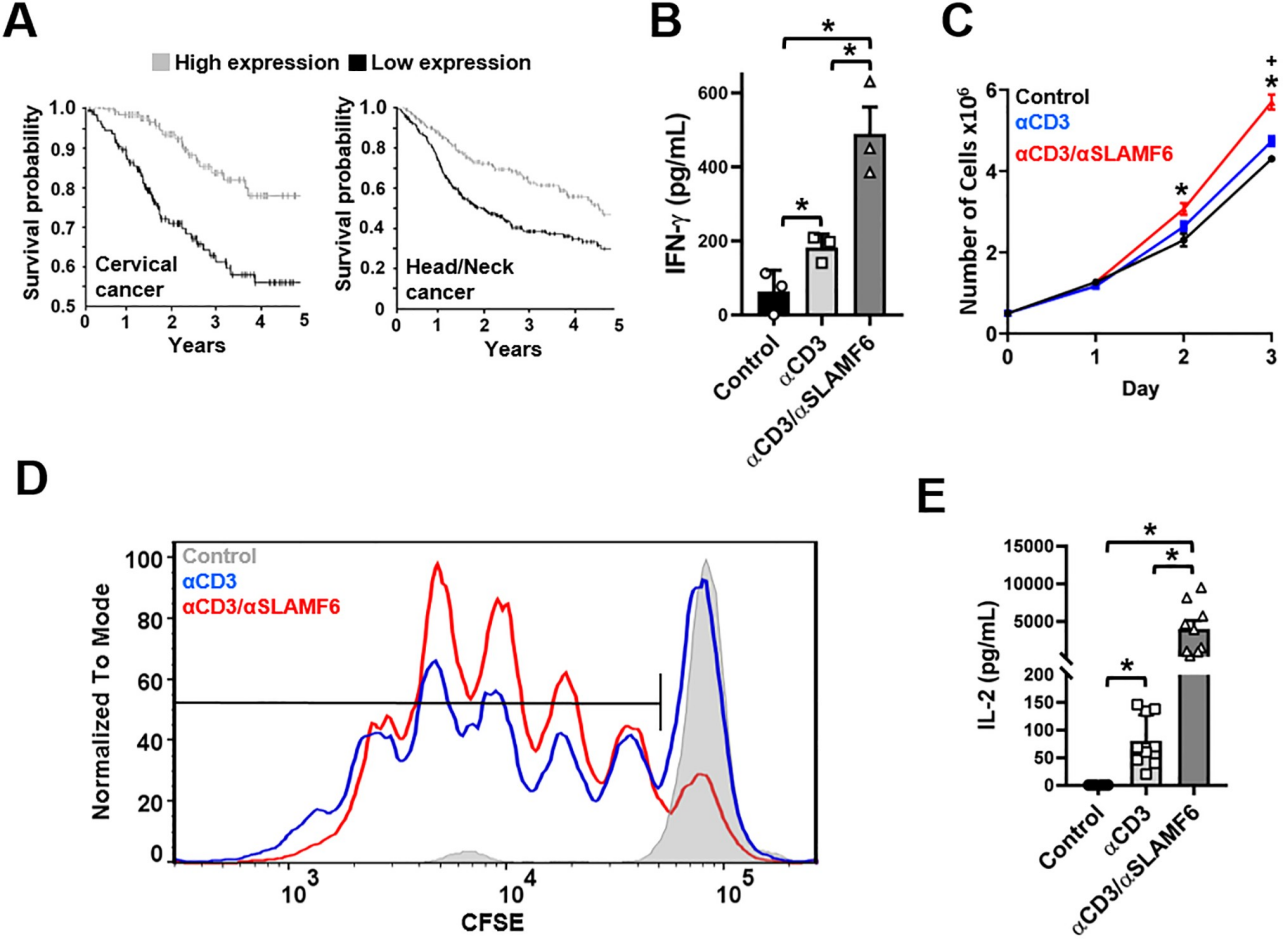
SLAMF6 antibody engagement leads to T cell activation. **(A)** Survival curves demonstrating that high expression of SLAMF6 in tumors is positively correlated with cancer survival. **(B)** Freshly isolated primary CD3^+^ T cells were cultured in the presence of αCD3 or αCD3 + αSLAMF6. After 48 hrs. the supernatant was harvested and IFN-γ levels were analyzed by ELSIA for two independent experiments (n = 2) with 3 technical replicates of one experiment shown. *p<0.05 for an unpaired student t-test **(C)** Jurkat T cells were cultured in the presence of αCD3 or αCD3 + αSLAMF6 for 72 hrs. Cell number was assessed by automated cell counting every 24 hrs. The experiment was done in triplicate (n = 3). *p<0.05 for αCD3 + αSLAMF6 compared to control and + p<0.05 for αCD3 compared to control for an unpaired student t-test. **(D)** Freshly isolated primary CD3^+^ T cells were stained with CFSE then cultured in the presence of immobilized αCD3 or αCD3 + αSLAMF6. After 120 hrs. the cells were assayed for FITC fluorescence for three independent experiments (n = 3). The data was analyzed for percent (%) of proliferating cells as depicted; αCD3 + αSLAMF6 had a 15–25% greater proliferation advantage over αCD3 alone conditions. **(E)** Also, the supernatant was harvested, and IL-2 levels were analyzed by ELSIA for three independent experiments (n = 3) with 3 technical replicates for each experiment shown together. *p<0.05 for an unpaired student t-test.

Limited data exist to support the role of SLAMF6 as a TCR stimulatory co-receptor [7, 15, 16]. Data mining from the cancer genome and human proteins atlases (http://www.proteinatlas.org/) revealed that high expression of SLAMF6 positively correlated with favorable outcomes for several solid malignancies, in particular cervical as well as head and neck cancers (Fig 1A). This lead us to ask if SLAMF6 engagement directly affects the TCR pathway. Thus, we treated primary human CD3^+^ T cells with anti-CD3 antibodies (αCD3) and with anti-SLAMF6 antibodies (αSLAMF6) and measured the levels of secreted IFN (interferon)-γ. Administration of αSLAMF6 and αCD3 together significantly increased IFN-γ levels compared to administration of αCD3 alone (Fig 1B). Moreover, culturing Jurkat T cells in the presence of αSLAMF6 and αCD3 together resulted in increased proliferation rate in comparison to culturing the cells with αCD3 alone (Fig 1C). Importantly, SLAMF6 expression remains stable after stimulation with αCD3 (S2 Fig). To further confirm our previous result, we performed CFSE proliferation assay with primary human CD3^+^ T cells. As shown, the presence of αSLAMF6 and αCD3 together resulted in increased proliferation rate in comparison to αCD3 alone (Fig 1D). Secreted IL-2 was also assayed, showing a dramatic increase in IL-2 that mirrored the proliferation results (Fig 1E).

### SLAMF6 expression increses TCR activation

**Fig 2:**
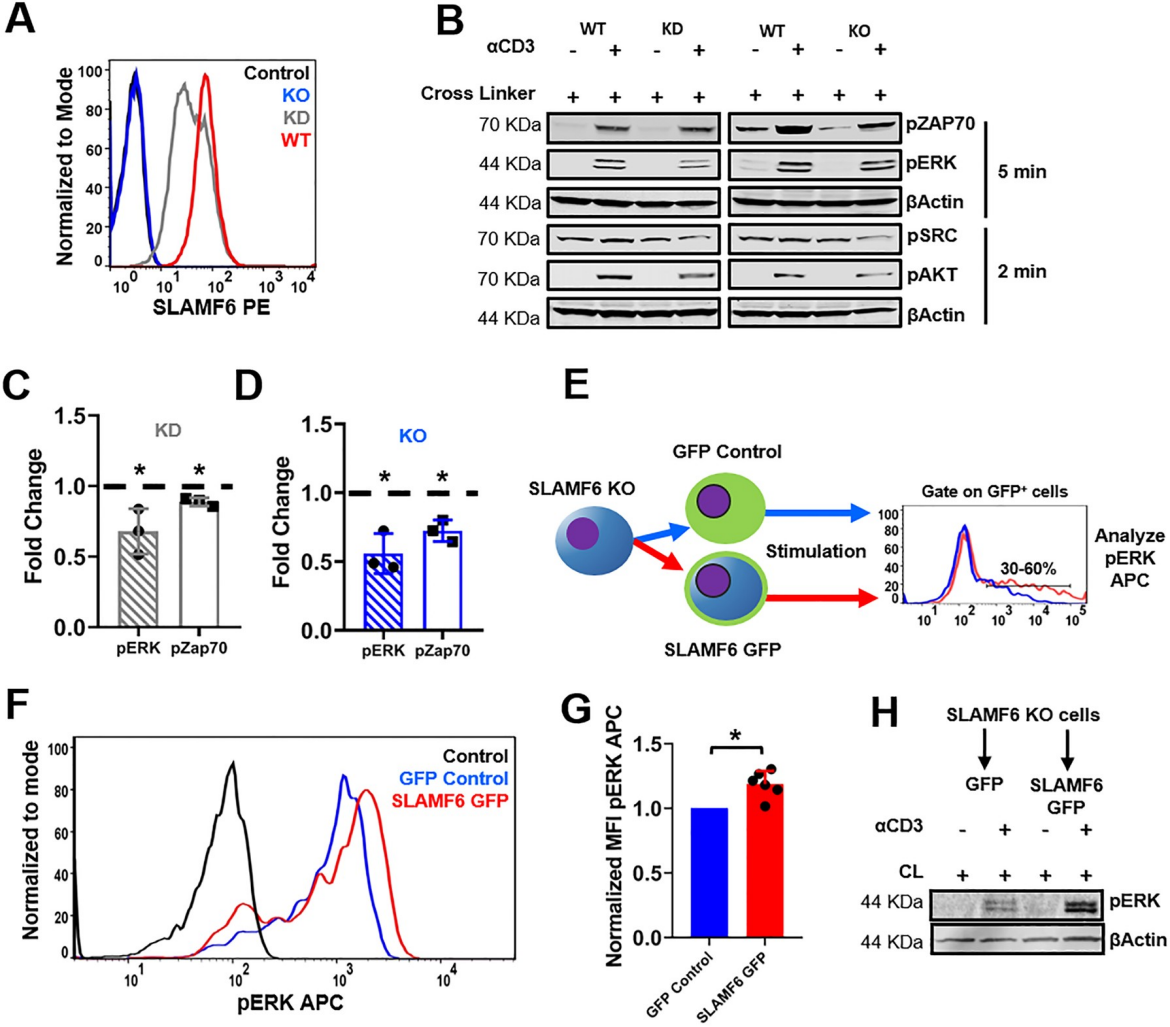
SLAMF6 knock down or knock out leads to a decrease in TCR activation. **(A)** Flow cytometry verification of SLAMF6 KO via CRISPR-Cas9 and SLAMF6 KD via shRNA. **(B)** WT Jurkat T cells, SLAMF6 KD and SLAMF6 KO were treated with αCD3 + cross linker and then stimulated at 37° C for 5 min. Blots were generated by lysing the cells, separating the lysates by tris-glycine PAGE and transferring to a nitrocellulose membrane. pZAP70, pERK, pSRC and pAKT were assessed. Quantification was given for three independent experiments (n = 3) for SLAMF6 KD Jurkat T cells **(C)** and SLAMF6 KO Jurkat T cells **(D)**. *p<0.05 for an unpaired student t-test. Further quantification of pSRC and pAKT can be found in S5 Fig. **(E)** A schematic of the flow cytometry assay used to rescue SLAMF6 function. SLAMF6 KO Jurkat T cells were transiently transfected with GFP control or SLAMF6 GFP. Cells were treated with αCD3 + cross linker, stimulated at 37° C for 5 min and subjected to Flow cytometry. Cell were gated on GFP positive cells and analyzed for pERK APC. **(F)** Representative curve for functional recue of SLAMF6 by flow cytometry. **(G)** Quantification for six independent functional recue of SLAMF6 by flow cytometry experiments (n = 6). *p<0.05 for an unpaired student t-test. **(H)** Functional recue of SLAMF6 by western blot. SLAMF6 KO cells were transiently transfected with GFP control (pEGFPN1 vector) or SLAMF6 GFP (SLAMF6 pEGFPN1 vector) then treated with αCD3 + cross linker and stimulated at 37° C for 5 min. Blot was generated and pERK were assessed.

To better define the role of SLAMF6 in T cells, we created an shRNA knock down (KD) as well as a CRISPR-Cas9 knock out (KO) of SLAMF6 in Jurkat T cell lines (Fig 2A). In both SLAMF6 KD and SLAMF6 KO cells, a decrease in ERK, ZAP70, SRC and AKT phosphorylation were observed (Fig 2B–2D & S3 Fig). Interestingly, the reduced levels of phosphorylated ERK and ZAP70 were more pronounced in the KO cells compared to the KD cells, suggesting gene dosage response. Alternative SLAMF6 inhibitory RNA sequences demonstrated the same results (S4 Fig). Furthermore, a control SLAMF7 shRNA failed to alter ERK and ZAP70 phosphorylation levels (S5 Fig). A transient re-expression of SLAMF6 GFP (Fig 2E) successfully rescued phospho-ERK responses (Fig 2F and 2G). Transient re-expression of SLAMF6 was not affected by the presence of Cas9 (S6 Fig). These results were validated via flow cytometry and by western blotting (Fig 2H).

### SLAMF6 clustering is required to augment T cell signaling

**Fig 3:**
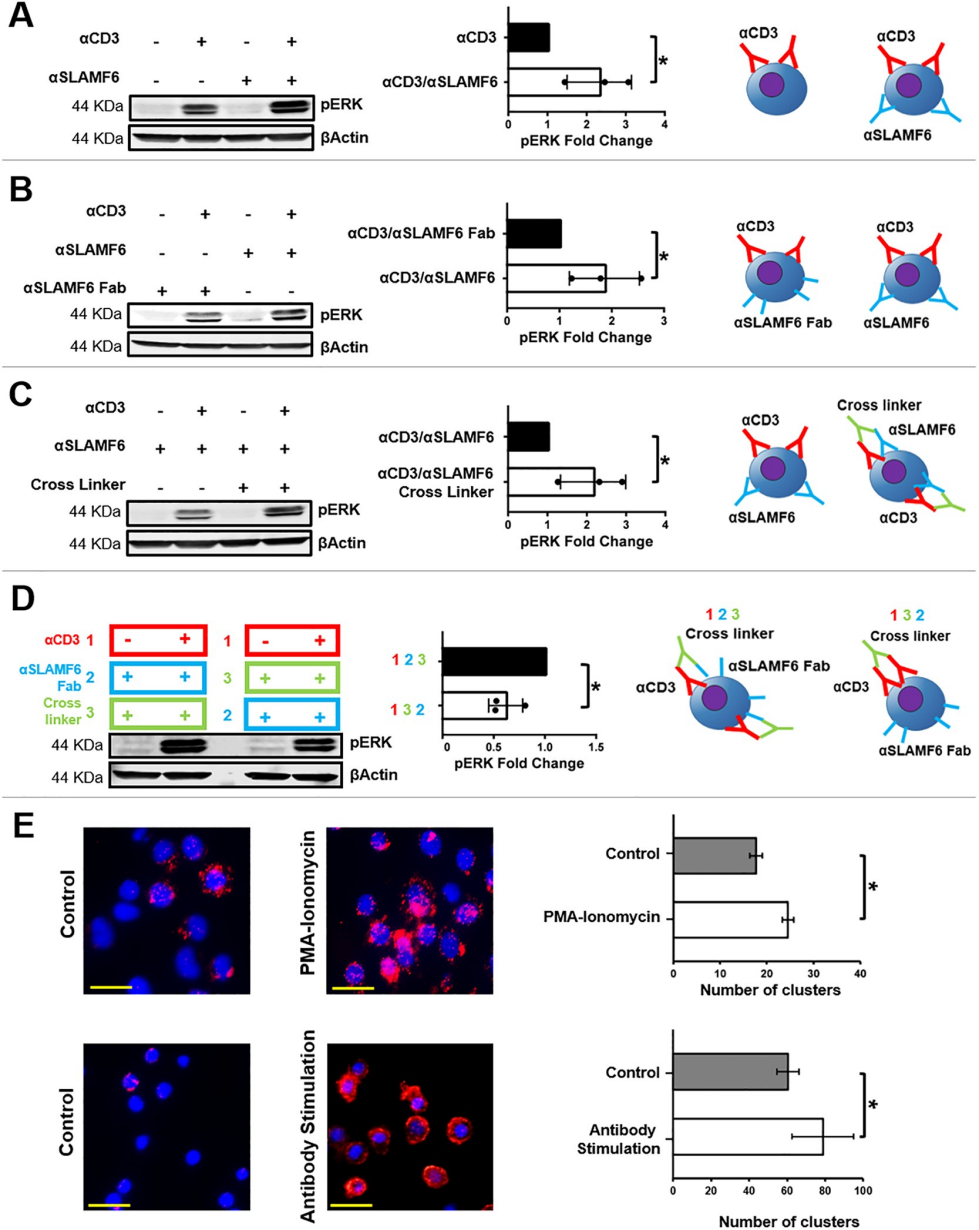
SLAMF6 clustering with the TCR increases T cell activation. **(A)** Jurkat T cells were treated with αCD3 or αCD3 + αSLAMF6 and then stimulated at 37° C for 5 min. Blots were generated by lysing the cells, separating the lysates by tris-glycine PAGE and transferring to a nitrocellulose membrane. pERK was assessed in three independent experiments (n = 3). *p<0.05 for an unpaired student t-test. **(B)** Jurkat T cells were treated with αCD3 + αSLAMF6 or αCD3 + αSLAMF6 Fab and then stimulated as above. Blots were generated assessing pERK in three independent experiments (n = 3). *p<0.05 for an unpaired student t-test. **(C)** Jurkat T cells were treated with αCD3 + αSLAMF6 or αCD3 + αSLAMF6 + cross linker and then stimulated. pERK was assessed in three independent experiments (n = 3). *p<0.05 for an unpaired student t-test. **(D)** Jurkat T cells were treated with αCD3 + αSLAMF6 Fab + cross linker or αCD3 + cross linker + αSLAMF6 Fab as delineated by color and number (cells were washed in between steps) and then stimulated. Blots were produced for pERK in three independent experiments (n = 3). *p<0.05 for an unpaired student t-test. **(E)** A proximity ligation assay (PLA) was performed on Jurkat T cells for SLAMF6 and the TCR. Cells were stimulated with PMA ionomycin at 37° C for 5 min, then fixed in 1% PFA for 10 min. Next, the cells were then blocked and treated with αCD3 + αSLAMF6 for 1 hr. at room temperature. This experiment was replicated in freshly isolated primary CD3^+^ T cells stimulated with immobilized αCD3 + αSLAMF6 for 120 hrs. The PLA assay was then performed as described by the manufactures protocol.

When Jurkat T cells were treated with αCD3 together with αSLAMF6, ERK phosphorylation was increased in comparison to cells that were treated with αCD3 alone (Fig 3A). To understand if SLAMF6-SLAMF6 *in-cis* clusters would enhance TCR activation, we treated the cells with αSLAMF6 or αSLAMF6 *Fab* and demonstrated reduced ERK phosphorylation with the latter (Fig 3B). To assess the contribution of TCR-SLAMF6 clustering to ERK phosphorylation, we added crosslinking antibodies to cells that were pre-treated with αCD3 and αSLAMF6 to demonstrate an increase of ERK phosphorylation upon crosslinking (Fig 3C). Moreover, we determined that crosslinking of αSLAMF6 *Fab* with the TCR also augmented ERK phosphorylation (Fig 3D). Lastly, we utilized a proximity ligation assay to demonstrate that endogenous TCR and SLAMF6 clustered upon stimulation with both Jurkat T cells (Fig 3E; Top) as well as freshly isolated primary CD3^+^ T cells (Fig 3E; Bottom).

### SLAMF6 traffics to the immunological synapse independent of its ectodomain

**Fig 4:**
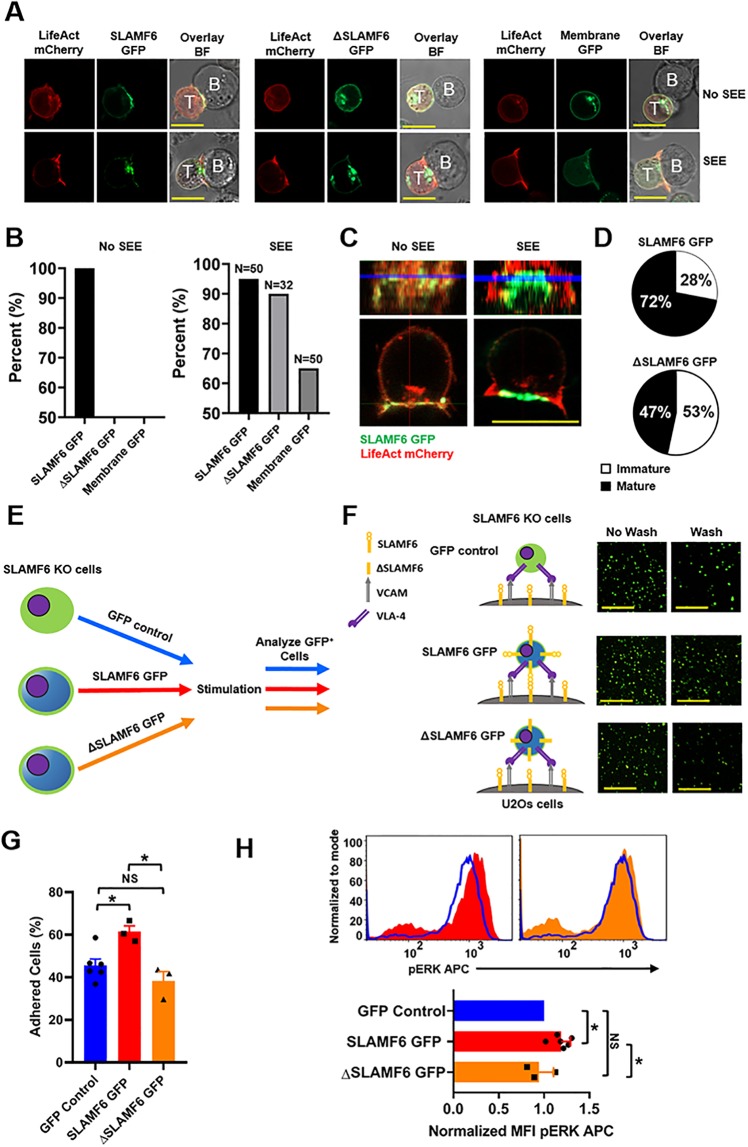
The SLAMF6 ectodomain is not required for SLAMF6 presence immunological synapse, but contributes to its co-receptor function. **(A)** Jurkat T cells were transfected with LifeAct mCherry and SLAMF6 GFP, ΔSLAMF6 or membrane GFP. Raji B cells (top row) or Raji B cells loaded with SEE (bottom row) were co-cultured with the transfected Jurkat T cells. Images are representative of at least 32 cells from a least two independent experiments. Scale bar is 10μm. Actin clearance was defined as a mature IS. If actin was not cleared from the IS the synapse was considered immature. Only images with actin clearance were considered for analysis of SLAMF6 presence in the IS when Raji B cells were loaded with SEE. **(B)** Quantification of SLAMF6 GFP, ΔSLAMF6 or membrane GFP at the point of contact between a Jurkat T cell and Raji B cell (left). Quantification of SLAMF6 GFP, ΔSLAMF6 GFP or membrane GFP at IS between a Jurkat T cell and a SEE loaded Raji B cell (right). **(C)** SLAMF6 localizes to the PSMAC in mature synapses. **(D)** The percent of mature and immature synapses for Jurkat T cells transfected with SLAMF6 GFP or ΔSLAMF6 GFP. **(E)** A schematic of the assay used to analyze SLAMF6 ectodomain function. **(F)** SLAMF6 KO jurkat T cells were transfected with GFP control, SLAMF6 GFP or ΔSLAMF6 and co-cultured with U2Os cells stably expressing SLAMF6 and VCAM in the presence of αCD3 for 20 min at 37°C. They were then imaged before and after four washes. Scale bar is 100μm. **(G)** Quantification of number of adhered cells for at least three independent experiments of SLAMF6 KO Jurkat T cells transfected with GFP control, SLAMF6 GFP or ΔSLAMF6 (bottom) (n = 3–6). *p<0.05 for an unpaired student t-test. **(H)** Representative histograms analyzing pERK APC for SLAMF6 KO Jurkat T cells (top). SLAMF6 KO Jurkat T cells were transiently transfected with GFP control, SLAMF6 GFP or ΔSLAMF6. Cells were treated with αCD3 + cross linker, stimulated at 37° C for 5 min, fixed permeabilized, stained and subjected to flow cytometry. Cells were gated on GFP positive cells and analyzed for pERK APC. Quantification of pERK for at least three independent experiments of SLAMF6 KO Jurkat T cells transfected with GFP control, SLAMF6 GFP or ΔSLAMF6 (bottom) (n = 3–6). *p<0.05 for an unpaired student t-test.

To discover if SLAMF6 is localized to the IS, we co expressed SLAMF6 green florescent protein (GFP) and Life-Act Cherry, a bio marker of actin polymerization, in Jurkat T cells. At base line, SLAMF6 was distributed almost exclusively to plasma membrane (S7 Fig), but upon conjugate formation with Raji B cells, SLAMF6 polarized toward the IS in all cells (Fig 4A left panel and 4B) as marked by actin clearance from that region (S8 Fig). Interestingly, this was independently of Staphylococcus Enterotoxin E (SEE) pre loading. Surprisingly, a version of SLAMF6 missing its ectodomain (ΔSLAMF6) also polarized toward the synapse, but only in the context of SEE (Fig 4A middle panel and 4B) suggesting that SEE-TCR downstream signals regulate SLAMF6 localization. ΔSLAMF6 trafficking to the IS was also, very dramatic in comparison to the membrane control GFP protein for which PAG (phosphoprotein associated with glycosphingolipid enriched micro domains) [17, 18], a known IS integral protein, was utilized (Fig 4A right panel and 4B). High resolution images confirmed that upon activation, SLAMF6 travels specifically to the peripheral rim of the synapse (Fig 4C) alongside the TCR [19, 20]. The consequences of the behavior of SLAMF6 were illustrated upon analyzing the percent of mature *vs*. immature synapses (S8 Fig) between Raji B cells and Jurkat T cells (Fig 4D). When T cells expressing wild type (WT) SLAMF6 came into contact with the Raji B cells, a greater number of cells demonstrated actin clearance from the IS compared to cells expressing ΔSLAMF6 (Fig 4D), suggesting that although the ectodomain of SLAMF6 was not required for its localization, it was necessary to promote synapse maturation via actin polarization. In order to better quantify the contribution of SLAMF6 to cellular adhesion we created a line of U20s osteosarcoma human epithelial cells that stably over expressed SLAMF6 and VCAM-1. We then introduced the either WT SLAMF6 or ΔSLAMF6 to the KO Jurkat T cells (Fig 4E) and adhesion of T cells to the U2Os cells was recorded. As shown in Fig 4F and quantified in Fig 4G, the ectodomain of SLAMF6 was absolutely required to promote adhesion via SLAMF6. To understand the contribution of SLAMF6 ectodomain to TCR activation by additional means, ERK phosphorylation was analyzed. We rescued the different versions of SLAMF6 to the KO Jurkat T cells (Fig 4E) and measured ERK phosphorylation by flow-cytometry after stimulation. As shown in Fig 4H, in the absence of the SLAMF6 ectodomain, phospho-ERK levels were reduced, suggesting that *outside-in* signaling via SLAMF6 contributed to ERK phosphorylation.

### SLAMF6 tyrosine 308 is crucial to its ability to promote adhesion

**Fig 5:**
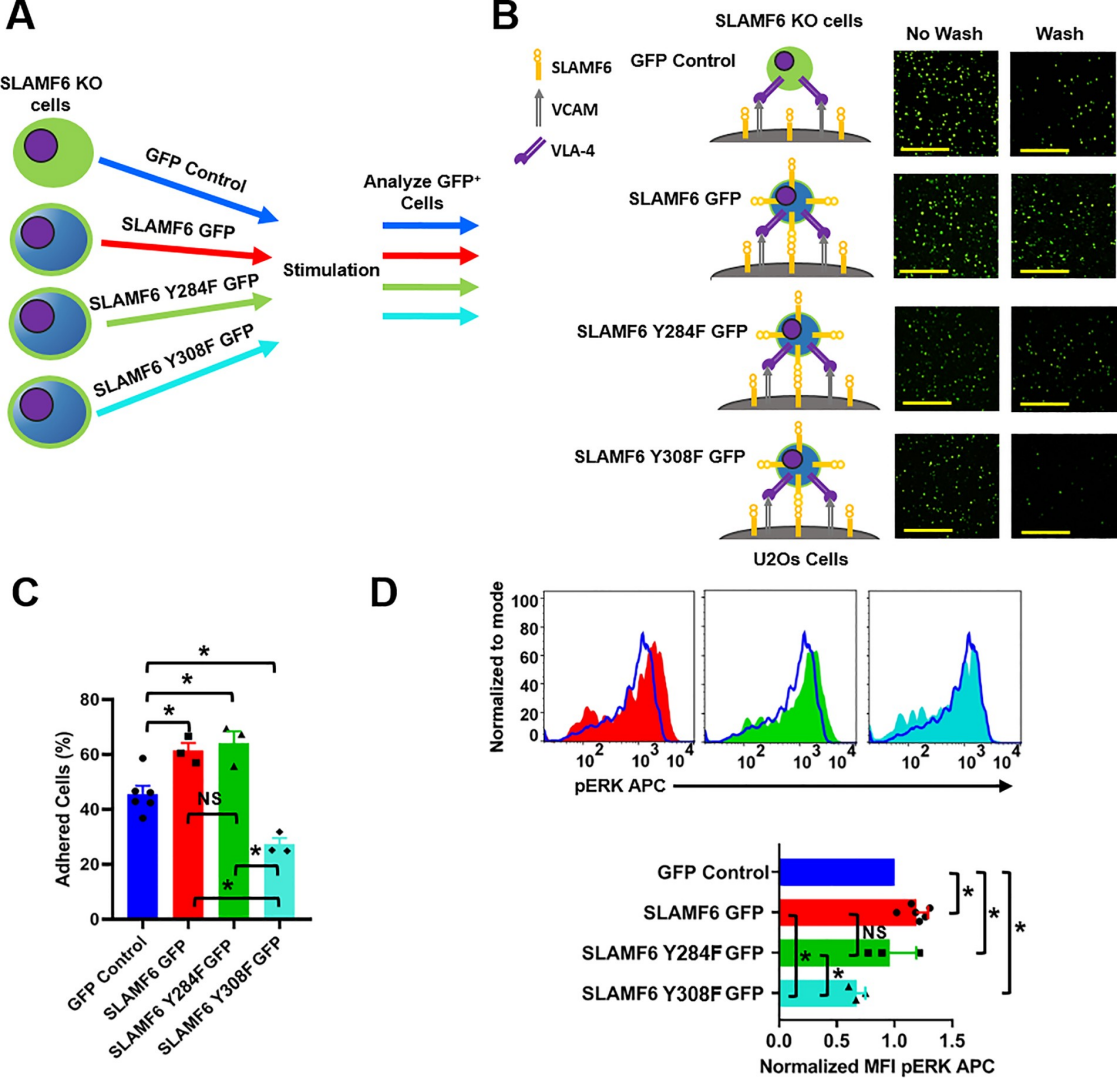
SLAMF6 tyrosine 308 is crucial for its ability to promote adhesion. **(A)** A schematic of the assay used to analyze SLAMF6 endodomain function. **(B)** SLAMF6 KO Jurkat T cells were transfected with GFP control, SLAMF6 GFP, SLAMF6 Y284F GFP and SLAMF6 Y308F GFP and co-cultured with U2Os cells stably expressing SLAMF6 and VCAM in the presence of αCD3 for 20 min at 37°C. They were then imaged before and after four washes. Scale bar is 100μm. **(C)** Quantification of number of adhered cells for at least three independent experiments of SLAMF6 KO Jurkat T cells transfected with GFP control, SLAMF6 GFP, SLAMF6 Y284F GFP and SLAMF6 Y308F GFP (bottom) (n = 3–6). *p<0.05 for an unpaired student t-test. **(D)** Representative histograms analyzing pERK APC for SLAMF6 KO Jurkat T cells t (top). SLAMF6 KO Jurkat T cells were transiently transfected with GFP control, SLAMF6 GFP, SLAMF6 Y284F GFP and SLAMF6 Y308F GFP. Cells were treated with αCD3 + cross linker, stimulated at 37° C for 5 min, fixed permeabilized, stained and subjected to flow cytometry. Cells were gated on GFP positive cells and analyzed for pERK APC. Quantification of pERK for at least three independent experiments of SLAMF6 KO Jurkat T cells transfected with GFP control, SLAMF6 GFP, SLAMF6 Y284F GFP and SLAMF6 Y308F GFP (bottom) (n = 3–6). *p<0.05 for an unpaired student t-test.

SLAMF6 holds two ITSM in its endodomain centered around tyrosines 284 and 308 [7–9]. As mentioned, these ITSM are known to bind to the adaptor proteins SAP and EAT-2 [7, 10]. Therefore, an additional flow-cytometry assay was carried out to investigate the manner by which the function of SLAMF6 is affected by the structure of its endodomain. SLAMF6 KO T cells were transiently re-expressed with WT SLAMF6 GFP or with phospho deficient versions of SLAMF6 (SLAMF6 Y284F GFP or SLAMF6 Y308F GFP) (Fig 5A) and an adhesion assay to U2Os cells expressing SLAMF6 and VCAM-1 (Fig 5B) was conducted. As shown in Fig 5B, and quantified in Fig 5C, SLAMF6 tyrosine 308 was crucial for cellular adhesions and also for ERK phosphorylation in stimulated cells (Fig 5D). This suggests that the function of the SLAMF6 ectodomain is achieved, at least in part, via tyrosine 308.

### SLAMF6 clustering increases Rap1 activation

**Fig 6:**
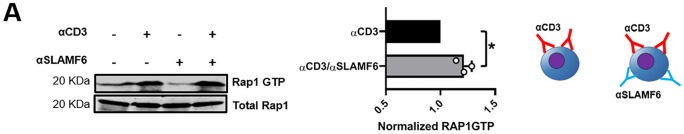
SLAMF6 clustering increases Rap1 activation. The amount of activated Rap1 was measured by glutathione *S*-transferase (GST) pull-down assay. Quantification is shown for at least three independent experiments. *p<0.05 for an unpaired student t-test.

Rap1 is a small GTPases mediating T cell adhesion and migration. To support a direct link between SLAMF6 signaling and T cell adhesion we measured the level of GTP loaded Rap1 in T cells that were stimulated with αCD3 and αSLAMF6 and demonstrated an increase in Rap1 GTP (Fig 6). Altogether we have shown that SLAMF6 regulates T cell activation and that. Moreover, that the ectodomain and endodomain of this protein contribute to TCR agonistic function through different mechanisms.

## Discussion

Studies have shown SLAMF6 to behave as an activating co-receptor on T cells [21, 22]. However, the molecular mechanisms have not yet been fully understood as to how SLAMF6 achieves this end. When we engaged SLAMF6 and the TCR with antibodies, a clear increase in TCR activity can be seen over controls that did not have antibody engaged SLAMF6. Also, we have shown that SLAMF6 requires clustering to function as a stimulatory co-receptor. When SLAMF6 was knocked down or knocked out, we have shown a decrease in TCR activity post TCR engagement. This occurred predominately at the distal node of the TCR pathway. Furthermore, we have newly elucidated that SLAMF6 is polarized to the IS during T cell-APC conjugation independent of its ectodomain and that SLAMF6 requires its ectodomain while in the IS to augment TCR activation. Lastly, we have shown that SLAMF6 tyrosine 308 and SLAMF6 ectodomain are important for TCR activation as well as integrin based adhesion.

Clustering is a key aspect for many co-receptors in augmenting the TCR pathway [4–6]. Our experiments have shown that SLAMF6 operates in this manner. Interestingly, when Jurkat T cells are treated with αSLAMF6 *Fab* less ERK phosphorylation is seen when compared to αSLAMF6, suggesting that SLAMF6 clusters are important regardless of their presence in the synapse during T-cell activation. This is perhaps due to need for SLAMF6 to engage SLAMF6 on the same cell which is an example of an *in-cis* clustering mechanism. Such a system has been elegantly described in NK cells by Wu *et al*. [23]. Moreover, this may allow for αSLAMF6 to engage the SLAMF6 pathway more robustly than SLAMF6-SLAMF6 ligation as the antibody promotes clustering. Furthermore, the likely reason for the SLAMF6 KO cells lack of signaling is that SLAMF6-SLAMF6 interactions are not allowed to occur during the stimulation process.

Our imaging analysis has demonstrated that SLAMF6 will be highly present in the IS and that SLAMF6 will traffic there independent of its ectodomain. However, SLAMF6 will only translocate to the point of contact between two cells that express SLAMF6 in a manner that is dependent on its ectodomain when the TCR is not engaged. Importantly, our flow-cytometry data indicates that although SLAMF6 is in the synapse it may not be able to provide co-receptor signals if it has not been engaged by another SLAMF6 molecule by the means of its ectodomain. Further, evidence for this is that when ectodomain deficient SLAMF6 is expressed, less mature synapses are able to form between the T cells and APC when compared to Jurkats T cells expressing WT SLAMF6. This is despite the fact that SLAMF6 may still be in the IS. Taken together our findings suggest a two-step model; SLAMF6 moves to the synapse during TCR engagement where it must then interact via its ectodomain with another SALMF6 molecule to augment TCR activation in an *outside-in-signaling* manner.

SLAMF6 and other SLAMF molecules in general depend on their association with SAP and other SH2 domain containing proteins to provide their function [9, 24–26]. SAP associates preferentially with ITSMs that contain the homology to TIYxxV/I/L/T, where x is any amino acid [27]. Therefore, SAP has been shown to associate with the second ITSM of SLAMF6 centered around tyrosine 308 [21, 28]. Herein, we have shown that changing of tyrosine 308 to phenylalanine will completely abolish the effects of SLAMF6 engagement leading to a reduction in TCR activation as well as activated T cell adhesiveness. Moreover, we have shown that SLAMF6 activates the GTPase Rap1, a master regulator of T cell adhesion, which acts to increase integrin activation [29]. Thus, connecting the importance of tyrosine 308 to integrin adhesion. Meaning that SLAMF6 may play an important role in leukocyte extravasation and perhaps T cell tumor infiltration by activing integrin and promoting firm adhesion.

SLAMF6 has been shown to have clinical significance [30–34]. In humans with X-linked lymphoproliferative (XLP) it has been demonstrated that blocking SLAMF4 and SLAMF6 via antibodies restores T cell function against B cell targets that also express SLAMF members [31]. Furthermore, SLAM plays a role in systemic lupus erythematosus (SLE). The *sleb1* locus corresponds to the SLAM genes and is located on chromosome 1 [35]. When investigated in 129Sv mice the sleb1 locus participates in the pathogenesis of SLE and that this was due to polymorphisms in *Slamf6* [35]. The consequence of these polymorphisms is enhanced signaling by the SLAMF6 receptor as well as changes in B and T cell functions that cause inflammatory symptoms [30, 35, 36]. In cancer, Eisenberg *et al*. has shown in great detail that administration of a soluble ectodomain of SLAMF6 increases the effector function CD8^+^ T cells against melanoma cells [34]. Furthermore, Yigit *et al*. has shown that the administration of SLAMF6 antibodies in combination with the BTK kinase inhibitor Ibrutinib has a positive effect on depleting leukemia cells from mice [33]. Given the aforementioned studies we have decided to proceed with SLAMF6 antibodies. This is likely due to our observation that SLAMF6 clustering, in proximity to the TCR, is important for its function in the TCR pathway. With an antibody based approach, drugs have the ability to dictate clustering via bi-specificity. This approach is not possible with soluble SLAMF6 ectodomain [34]. With these roles in human diseases in mind, it is imperative to further investigate the nature of SLAMF6 and how it behaves as an activating receptor in T cells.

To summarize, we have shown that SLAMF6 engagement can provide an important co-stimulatory signal to increase TCR activation. This wide-ranging role includes increasing cytokine secretion, proliferation, ERK and ZAP70 phosphorylation and cellular adhesion via Rap1 activation. Our findings suggest that during TCR engagement SLAMF6 will be brought to the IS independent of its ectodomain where it must be engaged by another SLAMF6 molecule via the ectodomain to realize its co-receptor function. SLAMF6 will then deliver a co-stimulatory signal into the T cell where the signal is dependent on tyrosine 308 and not tyrosine 284. By clarifying the mechanism by which SLAMF6 operates in the TCR pathway, we have provided a paradigm for ongoing studies of SLAMF6 in the treatment of diseases.

## Supporting information

S1 Fig Cartoon demonstrating the structure of SLAMF6. (TIF)

S2 Fig SLAMF6 expression remains constant after stimulation. Jurkat T cells were treated with αCD3 for 24 hrs. at 37°C. SLAMF6 expression was assed via flow cytometry. Representative histograms are shown (Left). Quantification was given for three independent experiments (n = 3) (right). No statistical significance was found when an unpaired student t-test was performed (p>0.05).
(TIF)

S3 Fig Additional quantification for SLAMF6 KD and KO western blots. WT Jurkat T cells, SLAMF6 KD and SLAMF6 KO were treated with αCD3 + cross linker and then stimulated at 37° C for 5 min. Blots were generated by lysing the cells, separating the lysates by tris-glycine PAGE and transferring to a nitrocellulose membrane. Quantification of pSRC and pAKT are shown. Blots are found in main text (Fig 2B).
(TIF)

S4 Fig An additional shRNA sequence was used to knock down SLAMF6. Representative histograms of SLAMF6 expression are shown (Left). Jurkat T cells were treated with αCD3 and cross linker for 5 min at 37°C. Blots were generated by lysing the cells, separating the lysates by tris-glycine PAGE and transferring to a nitrocellulose membrane. Representative blots shown (center). pZap70 and pERK were assessed in two independent experiments (n = 2) (right).
(TIF)

S5 Fig A shRNA sequence targeting SLAMF7 was used. Representative histograms of SLAMF7 expression are shown (Left). Jurkat T cells were treated with αCD3 and cross linker for 5 min at 37°C. Blots were generated by lysing the cells, separating the lysates by tris-glycine PAGE and transferring to a nitrocellulose membrane. Representative blots shown (center). pZap70 and pERK were assessed in two independent experiments (n = 2) (right).
(TIF)

S6 Fig Transient expression of SLAMF6 via nucleofection. WT Jurkat T cells (left) and SLAMF6 knock out (KO) Jurkat T cells (right).
(TIF)

S7 Fig Transient expression of SLAMF6 and LifeAct Cherry via nucleofection into WT Jurkat T cells. (TIF)

S8 Fig Cartoon demonstrating the formation of the Immunological synapse (IS) based on actin polarization. (TIF)

S9 Fig Raw unadjusted western blots presented in main figures. (TIF)
