# Supplementary figures and images for: SLAMF6 clustering is required to augment T cell activation
Source: PLoS One. 2019 Jun 14;14(6):e0218109. doi: 10.1371/journal.pone.0218109 (PMC6568412; doi:10.1371/journal.pone.0218109)

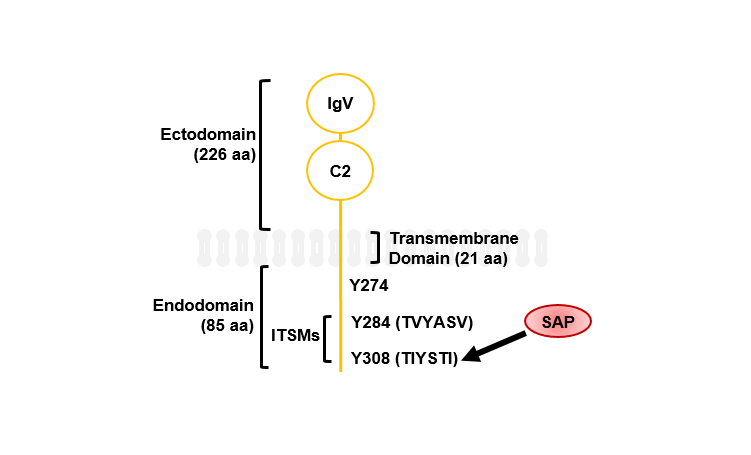

Supplement: S1 Fig — (TIF) [file pone.0218109.s001.tif]

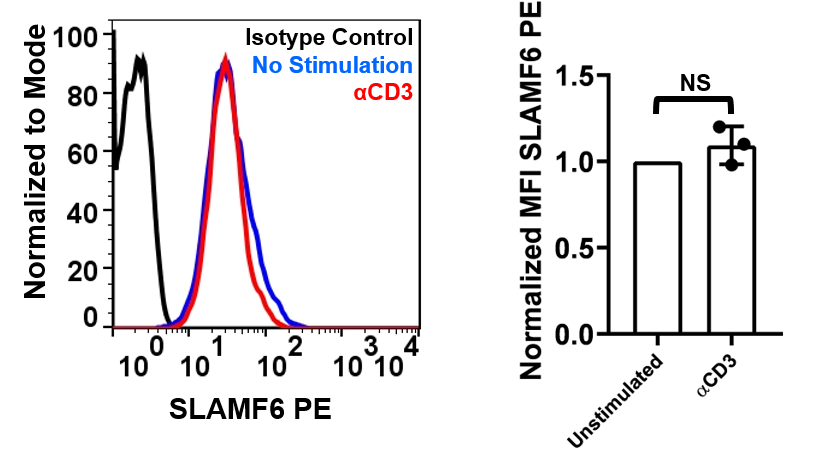

Supplement: S2 Fig — Jurkat T cells were treated with αCD3 for 24 hrs. at 37°C. SLAMF6 expression was assed via flow cytometry. Representative histograms are shown (Left). Quantification was given for three independent experiments (n = 3) (right). No statistical significance was found when an unpaired student t-test was performed (p>0.05). (TIF) [file pone.0218109.s002.tif]

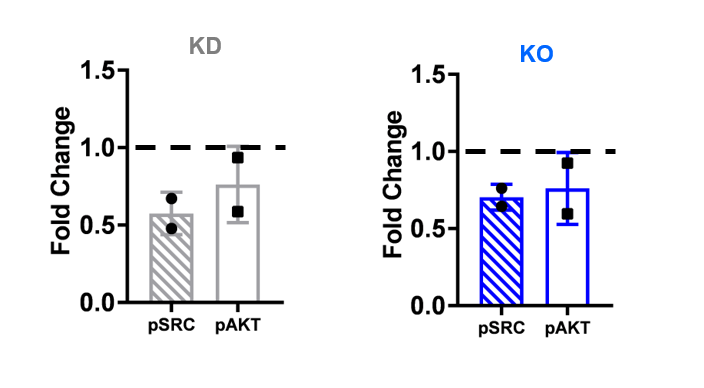

Supplement: S3 Fig — WT Jurkat T cells, SLAMF6 KD and SLAMF6 KO were treated with αCD3 + cross linker and then stimulated at 37° C for 5 min. Blots were generated by lysing the cells, separating the lysates by tris-glycine PAGE and transferring to a nitrocellulose membrane. Quantification of pSRC and pAKT are shown. Blots are found in main text (Fig 2B). (TIF) [file pone.0218109.s003.tif]

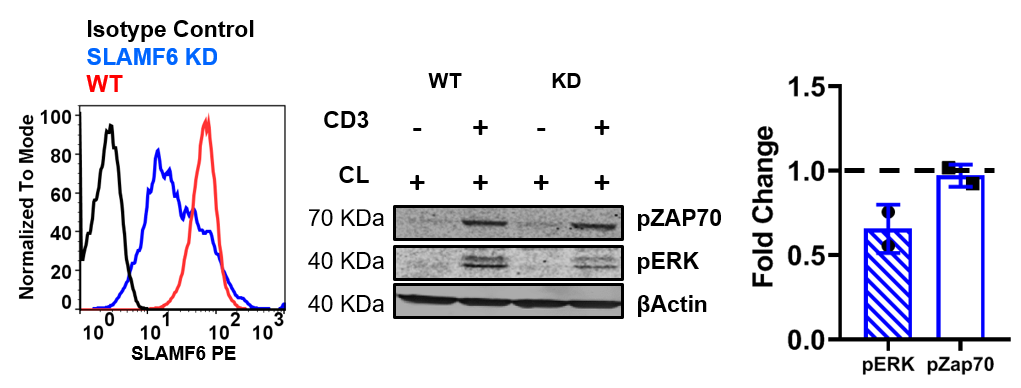

Supplement: S4 Fig — Representative histograms of SLAMF6 expression are shown (Left). Jurkat T cells were treated with αCD3 and cross linker for 5 min at 37°C. Blots were generated by lysing the cells, separating the lysates by tris-glycine PAGE and transferring to a nitrocellulose membrane. Representative blots shown (center). pZap70 and pERK were assessed in two independent experiments (n = 2) (right). (TIF) [file pone.0218109.s004.tif]

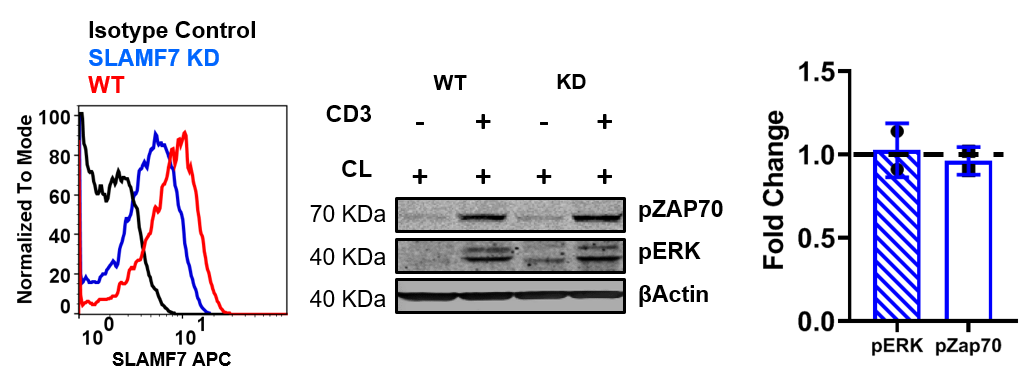

Supplement: S5 Fig — Representative histograms of SLAMF7 expression are shown (Left). Jurkat T cells were treated with αCD3 and cross linker for 5 min at 37°C. Blots were generated by lysing the cells, separating the lysates by tris-glycine PAGE and transferring to a nitrocellulose membrane. Representative blots shown (center). pZap70 and pERK were assessed in two independent experiments (n = 2) (right). (TIF) [file pone.0218109.s005.tif]

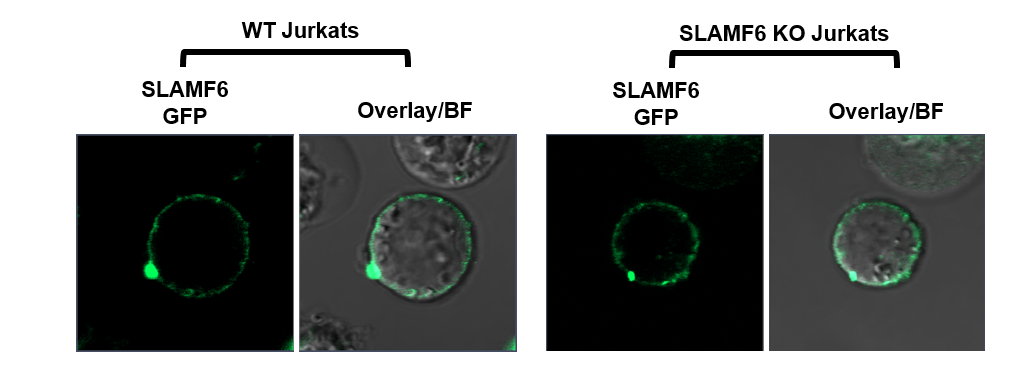

Supplement: S6 Fig — WT Jurkat T cells (left) and SLAMF6 knock out (KO) Jurkat T cells (right). (TIF) [file pone.0218109.s006.tif]

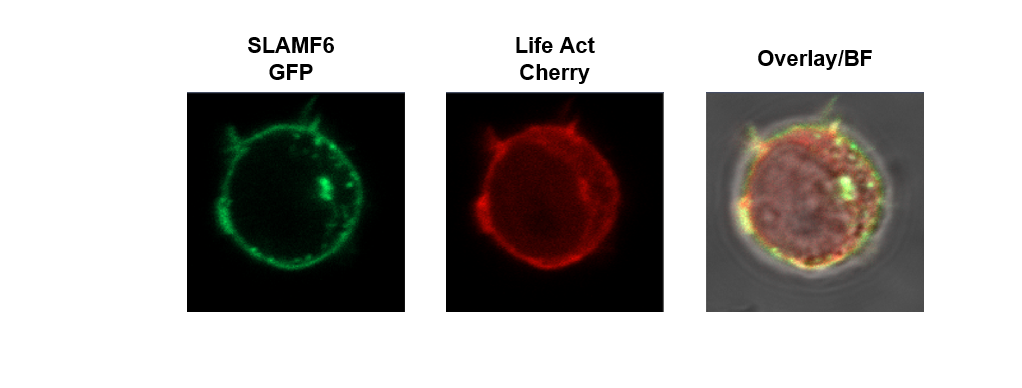

Supplement: S7 Fig — (TIF) [file pone.0218109.s007.tif]

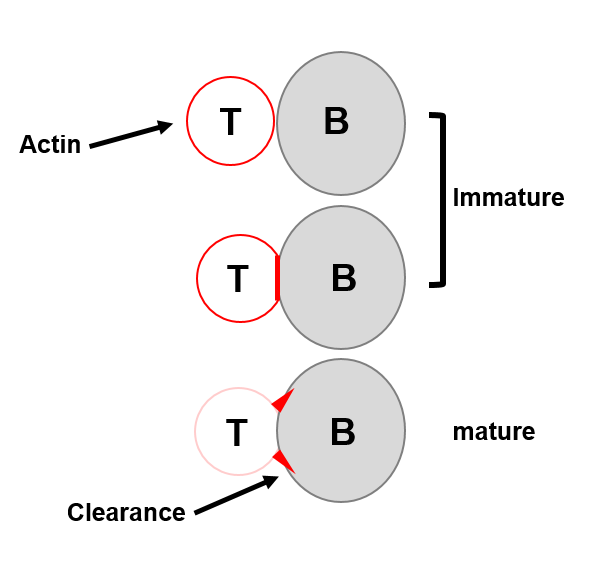

Supplement: S8 Fig — (TIF) [file pone.0218109.s008.tif]

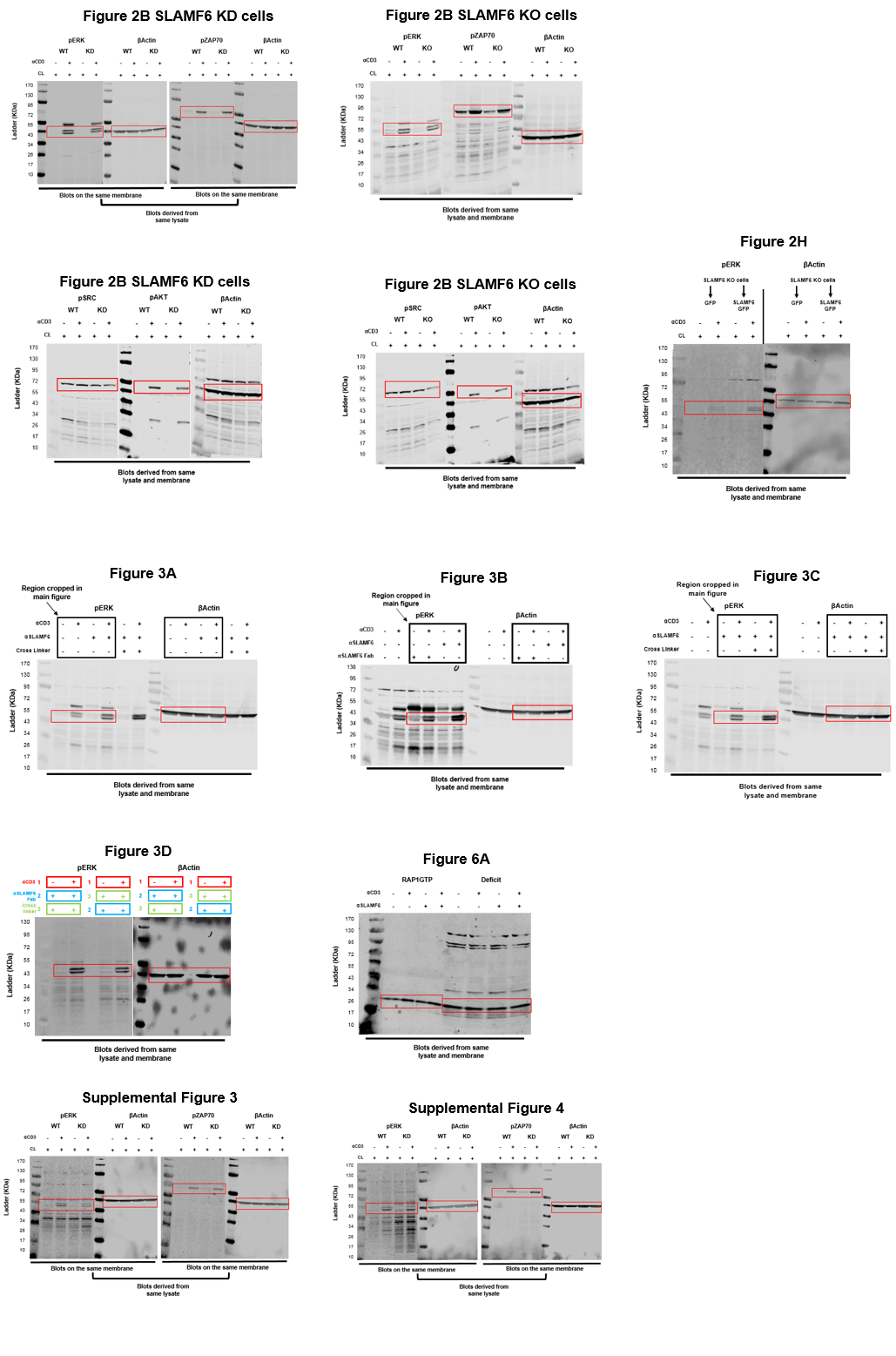

Supplement: S9 Fig — (TIF) [file pone.0218109.s009.tif]
